# Investigation of the Role of the TRPA1 Ion Channel in Conveying the Effect of Dimethyl Trisulfide on Vascular and Histological Changes in Serum-Transfer Arthritis

**DOI:** 10.3390/ph15060671

**Published:** 2022-05-27

**Authors:** István Z. Bátai, Ágnes Dombi, Éva Borbély, Ádám Fehér, Ferenc Papp, Zoltan Varga, Attila Mócsai, Zsuzsanna Helyes, Erika Pintér, Gábor Pozsgai

**Affiliations:** 1 Department of Pharmacology and Pharmacotherapy, Medical School, University of Pecs, Szigeti u. 12, H-7624 Pecs, Hungary; istvanzbatai@gmail.com (I.Z.B.); agi.borzsonyi@gmail.com (Á.D.); eva.borbely@aok.pte.hu (É.B.); zsuzsanna.helyes@aok.pte.hu (Z.H.); erika.pinter@aok.pte.hu (E.P.); 2 Molecular Pharmacology Research Group, Szentagothai Research Centre, University of Pecs, Ifjusag utja 20, H-7624 Pecs, Hungary; 3 Department of Anaesthesiology and Intensive Therapy, Clinical Centre, University of Pecs, Ifjusag utja 13, H-7624 Pecs, Hungary; 4 Department of Biophysics and Cell Biology, Faculty of Medicine, University of Debrecen, Egyetem ter 1, H-4032 Debrecen, Hungary; feher.adam@med.unideb.hu (Á.F.); pppfrnc@gmail.com (F.P.); veze@med.unideb.hu (Z.V.); 5 Department of Physiology, Faculty of Medicine, Semmelweis University, Tuzolto u. 37–47, H-1094 Budapest, Hungary; mocsai.attila@med.semmelweis-univ.hu

**Keywords:** dimethyl trisulfide, K/BxN serum-transfer arthritis, TRPA1 ion channel, plasma extravasation, fibroblast-like synoviocyte, cartilage destruction, patch clamp

## Abstract

Rheumatoid arthritis (RA) is one of the most prevalent autoimmune diseases. Its therapy is often challenging, even in the era of biologicals. Previously, we observed the anti-inflammatory effects of garlic-derived organic polysulfide dimethyl trisulfide (DMTS). Some of these effects were mediated by activation of the TRPA1 ion channel. *TRPA1* was mostly expressed in a subset of nociceptor neurons. We decided to investigate the action of DMTS in K/BxN serum-transfer arthritis, which is a relevant model of RA. *TRPA1* gene knockout (KO) and wild-type (WT) mice were used. The interaction of DMTS and TRPA1 was examined using a patch clamp in CHO cells. Arthritis was characterized by mechanical hyperalgesia, paw swelling, movement range of the ankle joint, hanging performance, plasma extravasation rate, myeloperoxidase activity, and histological changes in the tibiotarsal joint. DMTS activated TRPA1 channels dose-dependently. DMTS treatment reduced paw swelling and plasma extravasation in both *TRPA1* WT and KO animals. DMTS-treated *TRPA1* KO animals developed milder collagen deposition in the inflamed joints than WT ones. *TRPA1* WT mice did not exhibit significant cartilage damage compared to ones administered a vehicle. We concluded that DMTS and related substances might evolve into novel complementary therapeutic aids for RA patients.

## 1. Introduction

Rheumatoid arthritis (RA) affects 0.5–1% of the adult population. The disease potentially damages the joints of the hand by involving joint fusion, and severe disability can result if left untreated. Cardiovascular comorbidities and osteoporosis might contribute to the substantial deterioration of the quality of life [1]. The arsenal of the therapy of RA is ever-expanding. The present-day treatment relies on conventional synthetic, biological, and targeted synthetic disease-activity-modifying antirheumatic drugs. Despite expanding options, insufficient responders regarding single drug groups can tally at 40–50% of patients [2,3]. Adverse drug reactions are frequent among RA patients, with an incidence of 38.8% [4]. These conditions open the scape for research into novel antirheumatic drugs.

K/BxN serum-transfer arthritis is an exceptionally reproducible animal model of RA. The model resembles the systemic occurrence and autoimmune mechanism of human RA. The model is based on intraperitoneal administration of arthritic serum into target mice. Arthritic serum originates from offspring resulting from the crossing of two transgenic strains. The serum donor animals bear a mutation of the T-cell receptor and express the I-A^g7^ major histocompatibility complex class II molecule. The mice develop autoantibodies against glucose-6-phosphate isomerase [5]. Previously, we investigated the effects of sulfide donor GYY4137 in serum-transfer arthritis. We found that the effects were not mediated by sulfide, but by polysulfides being formed at the sites of inflammation. Polysulfides relieved mechanical hyperalgesia of the hind paws, improved clinical appearance of inflamed ankle joints, and mitigated cartilage damage in ankle joints in a TRPA1 ion-channel-dependent manner. Interestingly, mechanical hyperalgesia of the hind paws, myeloperoxidase activity of tibiotarsal joints, and subcutaneous chemokine concentration were elevated in mice genetically lacking functional TRPA1 channels [6]. Polysulfides have countless targets. They readily react with cysteine residues of proteins producing persulfides and coordinate with heme-bound iron [7,8]. The TRPA1 ion channel is activated by electrophilic substances reacting with critical cysteines of the channel protein. Polysulfides activate the ion channel this way [9]. Our data indicated that TRPA1 activation by GYY4137-derived polysulfides protected against mechanical hyperalgesia, edema formation, and cartilage destruction. Other protein targets of polysulfides are instead responsible for mechanical hyperalgesia and neutrophil infiltration. 

The TRPA1 ion channel is a member of the transient receptor potential family. TRPA1 channels are expressed in primary sensory nociceptor neurons and in some areas of the central nervous system [10,11,12,13]. Some papers have reported widespread extraneural expression of *TRPA1* (e.g., fibroblasts, alveolar epithelial cells, smooth muscle cells, melanocytes, keratinocytes, urothelium, astrocytes), but these accounts might be debated by others due to the potentially insufficient selectivity of antibodies used for immunochemistry [14]. TRPA1 ion channels are multimodal nonselective cation channels. They are activated by electrophilic chemicals, as mentioned above (e.g., allyl isothiocyanate, AITC; polysulfides, dimethyl trisulfide) [9,15]. Much fewer nonelectrophilic substances activate the channel via an orthodox agonist-binding pocket [16]. TRPA1 channels are sensitive to thermal and other stimuli [17]. 

Activation of TRPA1 leads to pain sensation and neurogenic inflammation due to calcitonin gene-related peptide and tachykinin release from nociceptor nerve endings [18]. This was substantiated by the mixed-function TRPA1 antagonist SZV 1287 inhibiting mechanical hyperalgesia, paw swelling, neutrophil accumulation, and microgliosis in the spinal cord dorsal horn. These effects were markedly reduced in *TRPA1* knockout mice [19]. In the long run, however, TRPA1 activation might initiate antihyperalgesic and anti-inflammatory processes in serum-transfer arthritis, as suggested by our previous data [6]. Such a protective effect of TRPA1 activation might rely on somatostatin release from peptidergic nociceptor nerve endings. Somatostatin activates SST4 receptors of spinothalamic and other neurons in the central nervous system [20]. 

Polysulfides are endogenously produced in mammals [21]. Endogenous polysulfides are reactive and short-lived [22]. Despite being potent modulators of protein function, they are not feasible therapeutics. Dimethyl trisulfide (DMTS) is an organic dialkyl polysulfide mostly present in garlic. It is used as a food additive. DMTS is chemically stable and possesses favorable pharmacokinetics, making the compound more suitable for pharmacological studies [23,24]. Earlier, we identified analgesic and anti-inflammatory effects of DMTS in heat injury, carrageenan-induced paw inflammation, and traumatic mononeuropathy [15,25,26]. Most of these effects were mediated by TRPA1 activation and subsequent somatostatin release followed by SST4 receptor activation. Surprisingly, DMTS exhibited a qualitatively different mechanism of action from that of sodium polysulfide in carrageenan-evoked paw inflammation. Polysulfide exerted its effects via TRPA1 and SST4, while mitigation of mechanical hyperalgesia and paw swelling by DMTS was SST4-dependent, but without the involvement of TRPA1. DMTS lowered myeloperoxidase activity in inflamed hind paws without the participation of TRPA1 or somatostatin [25]. Differences between the mechanisms of the two substances might be explained by their vastly different pharmacokinetics, enabling DMTS to reach targets unavailable for sodium polysulfide, and by chemically different interactions with protein cysteine residues. DMTS produces disulfide modifications rather than persulfide formation. Slightly differing thiol modifications might lead to a distinct conformational change and protein function [26]. 

Based on the beneficial pharmacokinetic characteristics and efficacy of DMTS, we decided to investigate its effect in the K/BxN serum-transfer arthritis model. Activation of TRPA1 ion channels by DMTS was demonstrated using a patch clamp in transfected Chinese hamster ovary (CHO) cells. The role of TRPA1 in serum-transfer arthritis was examined by the use of genetically modified mice. The mechanical pain threshold, volume of the hind paws, and grip endurance, as well as clinical appearance, myeloperoxidase activity, rate of plasma extravasation, and histological signs of arthritis in the ankle joints, were evaluated.

## 2. Results

### 2.1. DMTS Activated TRPA1 Ion Channels Concentration-Dependently

**Figure 1:**
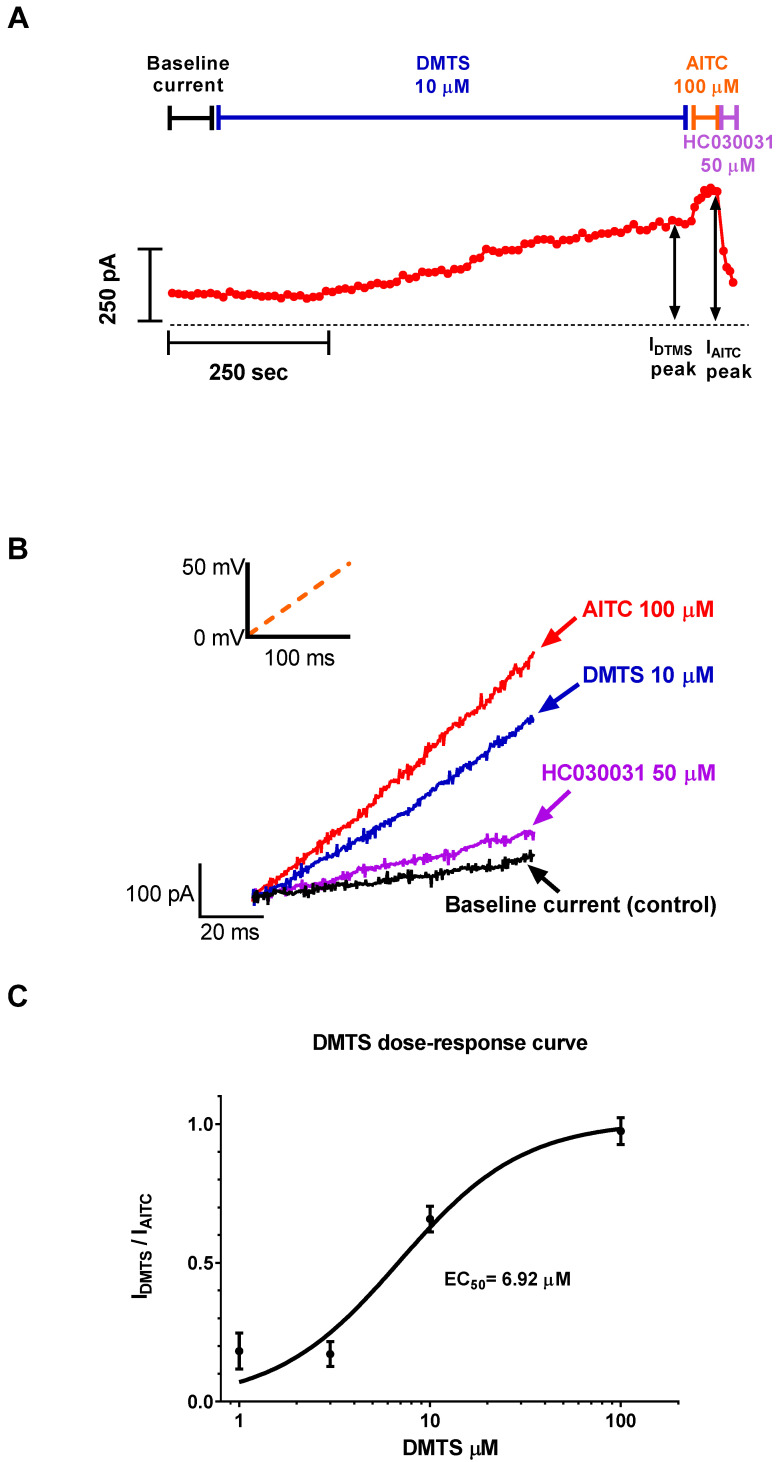
Effects of DMTS on *TRPA1* expressed in CHO cells. (**A**) Effects of DMTS (10 µmol/L) compared to that of AITC (100 µmol/L). The peak currents are shown at positive voltage (+50 mV). Dotted line indicates zero pA current. Individual current traces are presented as red circles (every second trace is shown). The baseline current (I_base_) represents the peak current measured before the addition of DTMS. The horizontal bars above indicate the period of time when the DMTS, AITC, and HC030031 (50 µmol/L) were applied. (**B**) Representative traces are depicted in this panel. Current increases caused by the activators are presented in blue (DMTS, 10 µmol/L) and red (AITC, 100 µmol/L). The effect of the inhibitor is in purple (HC030031 50 µmol/L). The black trace is the current measured in control, a Ca^2+^-free solution (i.e., baseline current). (**C**) Dose–response curve for DMTS. The vertical axis represents ionic current (I) evoked by DMTS normalized to the current induced by AITC, I_DMTS_/I_AITC_ = (I_DMTS_ peak-I_base_)/(IAITC peak-I_base_). Solid line indicates the best-fit Hill equation (see methods). The EC50 value was 6.92 µmol/L; error bars indicate SEM for n = 3 independent experiments; the Hill coefficient was 1.34.

CHO cells transfected with human *trpa1* were subjected to a manual patch clamp. Cells were voltage-clamped, and whole-cell currents were recorded. DMTS (10 µmol/L) induced currents comparable to those evoked by AITC (100 µmol/L). The current increased much slower in the case of DMTS than with AITC. The functionality of the ion channels was checked by applying the selective TRPA1 antagonist HC030031 at the end of the experiment (Figure 1A). A concentration–response curve was constructed using currents normalized to responses produced by AITC. The EC50 value was 6.92 µmol/L (Figure 1C).

### 2.2. DMTS Treatment Inhibited Paw Swelling and Arthritis Score Independent of TRPA1 Ion Channels

**Figure 2:**
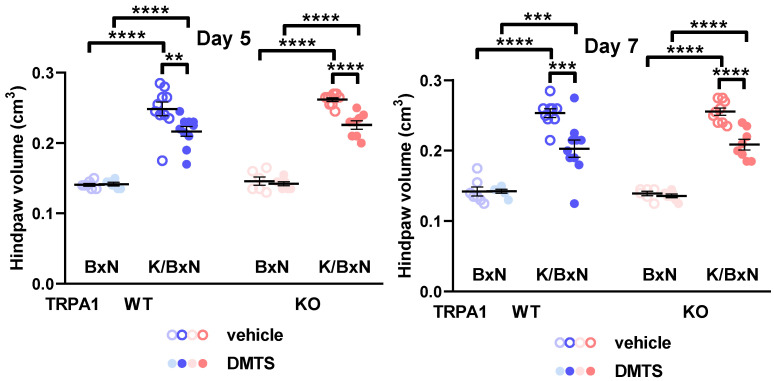
DMTS treatment ameliorated paw swelling in *TRPA1* WT and KO mice 5 and 7 days after the initiation of serum-transfer arthritis. Volume of the hind paws was measured using plethysmometry. Symbols indicate individual data points. Open symbols represent vehicle-treated animals, while solid symbols illustrate DMTS-treated ones. Horizontal lines indicate the mean, and whiskers show the SEM. One-way ANOVA and Holm–Sidak’s test: n = 6–10. ** *p* < 0.01, *** *p* < 0.001, **** *p* < 0.0001 vs. indicated group.

**Figure 3:**
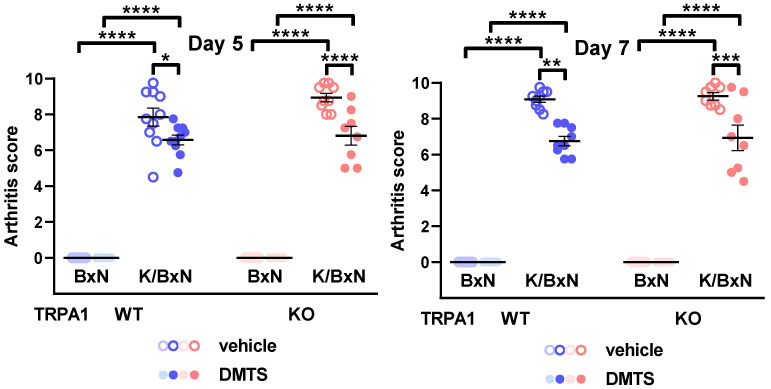
DMTS treatment reduced arthritis score of *TRPA1* WT and KO mice 5 and 7 days after the administration of arthritogenic serum. The score represents swelling and redness of the hind paws, as well as passive mobility of the tibiotarsal joints. Symbols indicate individual data points. Open symbols represent vehicle-treated animals, while solid symbols illustrate DMTS-treated ones. Horizontal lines indicate mean and whiskers show SEM. One-way ANOVA and Holm-Sidak’s test: n = 6–10. * *p* < 0.05, ** *p* < 0.01, *** *p* < 0.001, **** *p* < 0.0001 vs. indicated group.

The parameters of the serum-transferred mice were detected on days 5 and 7 after the serum injections. Daily DMTS treatment (125 µmol/L, i.p.) had no effect on any examined parameters in nonarthritic animals treated with BxN serum. Administration of K/BxN serum increased the mechanical sensitivity, volume, and arthritis score of the hind paws compared to the nonarthritic control. The grip endurance of the mice was readily deteriorated by the arthritogenic serum treatment compared to the BxN group (Supplementary Figures S1 and S2 and Figure 2 and Figure 3). Swelling of the hind paws was ameliorated by DMTS at 5 and 7 days after the initiation of arthritis in mice injected with K/BxN serum. The effect occurred in both *TRPA1* WT and KO animals (n = 6–10; Figure 2). Similarly, the arthritis score, consisting of redness, swelling and passive mobility of the ankle joint, was reduced by DMTS application on days 5 and 7. The protective effect manifested in *TRPA1* WT and KO animals (n = 6–9; Figure 3). DMTS treatment influenced neither mechanical sensitivity of the hind paws nor grip stamina of the animals (Supplementary Figures S1 and S2).

### 2.3. DMTS Reduced the Actual Rate of Plasma Extravasation in a TRPA1-Independent Manner

**Figure 4:**
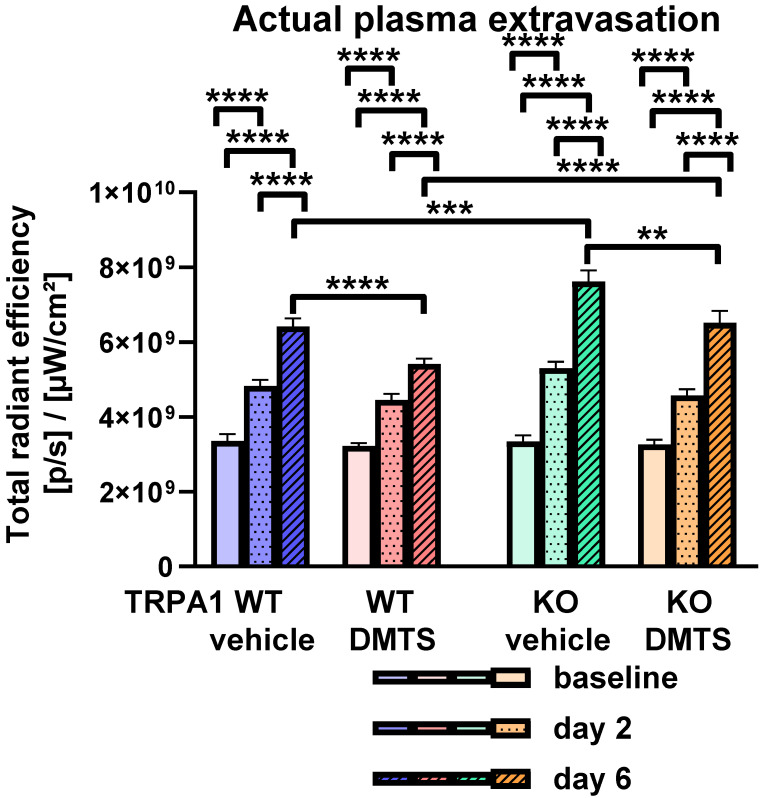
DMTS treatment lowered plasma extravasation rate 6 days after the injection of arthritogenic serum in *TRPA1* WT and KO mice. The rate of plasma extravasation was detected by fluorescent imaging of IR676. Solid bars represent baseline values, dotted bars show fluorescence at day 2, and striped bars indicate plasma extravasation at day 6. One-way ANOVA and Holm-Sidak’s test: n = 16–20. ** *p* < 0.01, *** *p* < 0.001, **** *p* < 0.0001 vs. indicated group.

The actual rate of plasma extravasation in the hind paws was measured using fluorescent imaging of micellated IR676 dye 2 and 6 days after challenge with K/BxN serum. This method detected the velocity of extravasation in a 20 min interval. Serum-transfer arthritis elevated the plasma extravasation both 2 and 6 days after serum treatment compared to the baseline values in *TRPA1* WT and KO animals administered a vehicle or DMTS (Figure 4). The extravasation values increased during the examined time span. On day 6, DMTS reduced the rate of plasma extravasation in *TRPA1* WT and KO mice compared to vehicle-treated ones (Figure 4). Interestingly, *TRPA1* KO vehicle-treated animals exhibited a larger extravasation rate on day 6 compared to *TRPA1* WT vehicle-treated ones (Figure 4). A similar difference occurred between *TRPA1* WT and KO mice injected with DMTS. 

MPO activity of the hind paws was detected using luminescent imaging of luminol 2 and 6 days after the initiation of arthritis. *TRPA1* WT and KO mice with serum transfer arthritis showed elevated MPO activity 2 and 6 days after challenge irrespective of vehicle or DMTS treatment compared to the baseline enzyme activity. MPO activity values were much smaller on day 6 than on day 2 (Supplementary Figure S3). DMTS treatment had no effect on MPO activity. *TRPA1* KO vehicle-treated animals had higher MPO activity than *TRPA1* WT vehicle-treated ones (Supplementary Figure S3). 

### 2.4. DMTS-Treated TRPA1 KO Animals Exhibited Milder Collagen Deposition in the Ankle Joints Than WT Ones

**Figure 5:**
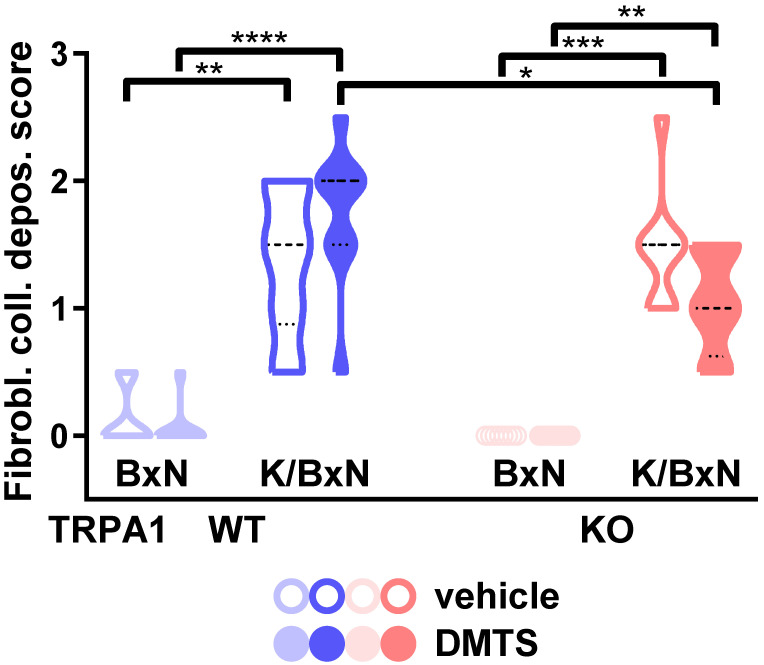
Amounts of fibroblasts and collagen deposition were smaller in arthritic *TRPA1* KO mice treated with DMTS than in WT ones. The amounts of fibroblasts and collagen deposition were scored on decalcified hematoxylin and eosin-stained slides of tibiotarsal joints of hind legs. Width of violin plots corresponds with frequency density of the data. Open violin plots represent vehicle-treated groups; solid ones indicate animals injected with DMTS. Dashed lines show medians and dotted lines display quartiles. Kruskal–Wallis test followed by Dunn’s test: n = 8–10. * *p* < 0.05, ** *p* < 0.01, *** *p* < 0.001, **** *p* < 0.0001 vs. indicated group.

**Figure 6:**
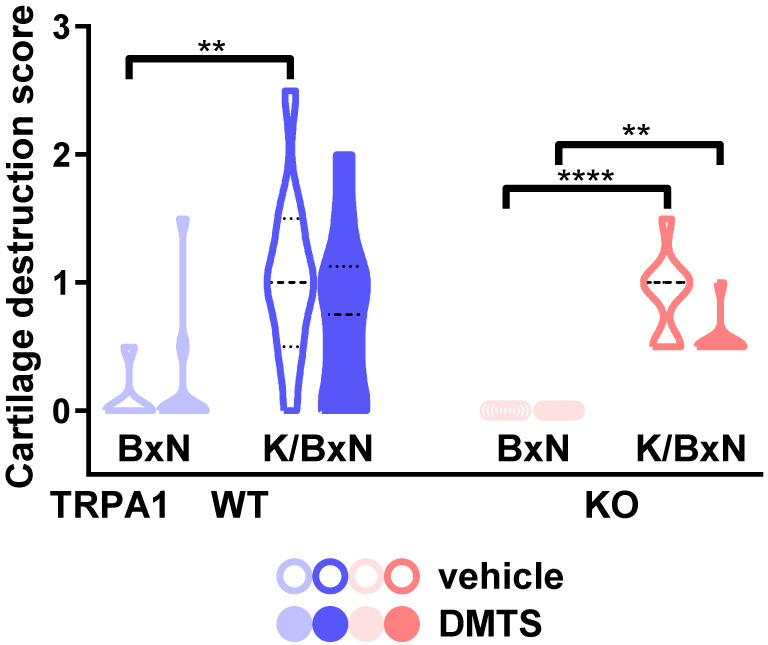
Cartilage-destruction score did not increase significantly in arthritic *TRPA1* WT DMTS-administered mice compared to nonarthritic control ones. Cartilage damage was scored on decalcified hematoxylin and eosin-stained slides of ankle joints of hind legs. Width of violin plots indicates frequency density of the data. Open violin plots represent vehicle-treated groups; solid ones show ones administered DMTS. Dashed lines show medians and dotted lines display quartiles. Kruskal–Wallis test followed by Dunn’s test: n = 8–10. ** *p* < 0.01, **** *p* < 0.0001 vs. indicated group.

Seven days after the administration of K/BxN serum, tibiotarsal joints were harvested and processed histologically. Four characteristics were scored: number of infiltrating mononuclear cells, synovial proliferation, collagen deposition, and cartilage destruction. All four parameters scored were increased in arthritic mice compared to BxN-injected controls regardless of genotype or DMTS treatment, except for cartilage destruction in the *TRPA1* WT DMTS-treated group (Supplementary Figures S4 and S5 and Figure 5 and Figure 6). The amounts of fibroblast-like synoviocytes (FLS) and collagen deposition were found to be smaller in *TRPA1* KO animals administered DMTS compared to *TRPA1* WT ones (Figure 5). However, the collagen-deposition data of arthritic DMTS-treated *TRPA1* WT and KO animals did not differ from those of the respective vehicle-treated ones. As mentioned above, the cartilage-destruction score of the *TRPA1* WT arthritic mice in the DMTS group did not increase significantly compared to the nonarthritic animals. It must be pointed out that the cartilage-destruction score of the K/BxN serum-injected *TRPA1* WT animals administered DMTS did not differ from that of vehicle-treated ones (Figure 6). 

## 3. Discussion

Our study confirmed that DMTS is a potent agonist of TRPA1 channels [15,27]. We demonstrated that DMTS mitigated edema formation and plasma extravasation in the K/BxN serum-transfer arthritis model as well. On the other hand, DMTS treatment had no effect on either mechanical hyperalgesia of the inflamed hind paws or the hanging performance of the animals. DMTS administration affected neither the number of neutrophil granulocytes nor that of mononuclear cells. Synovial hyperplasia was not relieved by DMTS application. The amount of FLS in tibiotarsal joints was reduced by DMTS in *TRPA1* KO animals, but was unaltered in *TRPA1* WT ones. No cartilage destruction developed in DMTS-treated *TRPA1* WT mice compared to nonarthritic controls, in contrast to *TRPA1* KO animals, which exhibited marked cartilage damage. 

Sulfide compounds are extremely promiscuous. They might react with cysteine residues of a vast number of proteins. It is sure that the transmission of sulfide effect is not limited to TRPA1 activation. Albeit, TRPA1 has a dominant role in some sulfide effects, illustrated by a lack of these effects in *TRPA1* KO mice. Many researchers published an activation of TRPV1 ion channels by sulfide. TRPV1 channels are similar to TRPA1 ones. TRPA1 and TRPV1 are co-expressed in primary nociceptor neurons. TRPV1 channels are more abundantly expressed in these neurons. Activation of TRPV1 induces similar, often identical cellular responses to TRPA1 activation (e.g. calcium influx and neuropeptide release). TRPV1 activation by sulfide was demonstrated in primary cultures of colorectal cancer cells by ratiometric calcium imaging. Calcium responses were blunted by TRPV1 antagonists [28]. Similar data were produced in human dental pulp stem cells [29]. NaHS stimulated gastric acid secretion in rats. The response was mitigated by a TRPV1 antagonist [30]. NaHS sensitized carotid sinus baroreceptors in the rat. The effect could be reduced by TRPV1 antagonist treatment [31]. Based on these data, TRPV1 activation by DMTS could contribute to our present findings. Earlier, we performed experiments on CHO cells expressing TRPA1 and TRPV1 ion channels. Calcium influx was detected by fluo-4 fluorescence using flow cytometry. In our hands DMTS did not induce significant responses either in TRPV1-expressing cells or non-transfected CHO cells, but in cells possessing TRPA1 channels [15]. Further investigating TRPA1-expressing CHO cells, we found that Na_2_S nonahydrate (a sulfide donor similar to NaHS) only produces calcium influx in supraphysiological concentration. EC_50_ was close to 10 mmol/L. These responses occurred in non-transfected CHO cells, indicating mitochondrial dysfunction rather than calcium influx across the plasma membrane [6]. 

Previously, we examined the effect of sulfide donor GYY4137 in K/BxN serum-transfer arthritis [6]. Inorganic sodium polysulfide created at the sites of inflammation had a markedly different influence on the arthritic process compared to that of DMTS. Polysulfide originating from GYY4137 exhibited distinct effects in *TRPA1* WT and KO mice. Mechanical hyperalgesia, the arthritis score, and damage to cartilage were mitigated in *TRPA1* WT subjects. Mechanical hyperalgesia, plasma extravasation, and MPO activity were exacerbated in *TRPA1* KO mice. No such disparity was seen between *TRPA1* WT and KO animals after DMTS treatment. DMTS did not feature any proinflammatory effects in *TRPA1* KO mice. An elevated rate of plasma extravasation was detected in *TRPA1* KO animals. However, this was not only present in DMTS-treated mice, but also in vehicle-treated *TRPA1* KO mice, and DMTS lowered the extravasation rate compared to the vehicle. Plasma extravasation was reduced by DMTS in *TRPA1* WT mice as well. DMTS did not abate mechanical hyperalgesia or neutrophil cell accumulation in the inflamed joints. DMTS attenuated vascular inflammation characterized by paw volume, arthritis score, and plasma extravasation rate in both *TRPA1* WT and KO mice. We collected similar data earlier in carrageenan-induced paw inflammation [25]. The action of DMTS was found to rely on somatostatin from peptidergic nociceptor nerve endings. The proposed triggers of peptide release were either activation of CaV 3.2 calcium channels or inhibition of KV 4.3 potassium channels by sulfide compounds [25]. 

Somatostatin is a cyclic peptide. It can be released from peptidergic nociceptor neurons. Somatostatin was released from nerve endings of isolated murine skin upon application of sodium polysulfide due to the activation of TRPA1 ion channels [6]. Somatostatin enters the systemic circulation and exerts analgesic and anti-inflammatory effects via sst4 receptors [32]. Our earlier data indicated that a reduction in vascular inflammation by DMTS was mediated by sst4 activation, but not by TRPA1 activation [25]. 

We detected a larger number of FLS and heavier collagen deposition in arthritic *TRPA1* WT mice of the DMTS group than in their KO counterparts. This coincided with our previous findings in the serum-transfer arthritis model [6]. TRPA1 ion channels are expressed in mesenchymal cells; e.g., in FLS [33]. FLS contribute to K/BxN arthritis by releasing chemokines, cytokines, and matrix metalloproteinases. Many papers have corroborate the proinflammatory role of TRPA1 activation in fibroblasts. Lipopolysaccharide (LPS) stimulation increased *TRPA1* expression in human osteoarthritic FLS. Cytokine release from FLS and cartilage destruction were mitigated by a TRPA1 antagonist [34]. These data can be put in line with our present results in *TRPA1* WT mice regarding the elevated number of FLS and more severe collagen deposition. Others attributed protective outcomes to TRPA1 activation in mesenchymal cells. The TRPA1 agonist allyl isothiocyanate ameliorated the expression of α-SMA [35]. Activation of TRPA1 by polygodial in rheumatoid arthritis FLS led to necrosis of the cells and reduced proliferation [36]. The synthetic cannabinoid WIN55,212-2 mesylate activated TRPA1. In rheumatoid arthritis FLS, WIN55,212-2 mesylate exerted dose-dependent effects. A smaller dose inhibited inflammatory cytokine and matrix metalloproteinase 3 secretion. A larger dose had similar effects on cytokine release, but instead stimulated metalloproteinase release [37]. Sulfide-releasing substances have a long history of anti-inflammatory activity in FLS. S-propyl cysteine inhibited cytokine, oxygen radical, and MMP release from MH7A human FLS via the Keap1/Nrf2 signaling pathway [38]. Similar findings were produced with NaHS in osteoarthritic and RA FLS [38,39,40]. Diallyl trisulfide, a garlic-derived compound similar to DMTS, mitigated TNF-α-evoked cytokine release in FLS isolated from animals with collagen-induced arthritis [41]. Such mechanisms might explain the lower FLS density and collagen deposition detected in *TRPA1* KO animals in our study. 

In concert with our previous findings in the K/BxN serum-transfer arthritis model, DMTS-treated arthritic *TRPA1* WT animals did not exhibit elevated cartilage destruction compared to nonarthritic ones, whereas *TRPA1* KO mice did [6]. MMPs are widely expressed in fibroblasts, and contribute to matrix destruction in cartilage tissue [42]. TRPA1 activation might relieve pathological cartilage destruction by inhibiting MMP release from mesenchymal cells. TRPA1 activation by cannabidiol reduced MMP3 liberation from FLS [43]. Similar results were obtained in human RA FLS with WIN55,212-2 mesylate [37]. On the other hand, several reports implicated the damaging outcome of TRPA1 activation on cartilage. A TRPA1 antagonist diminished cartilage destruction in LPS-induced knee arthritis in rats [34]. *TRPA1* is expressed in chondrocytes as well. Activation of the ion channel in chondrocytes was proposed to harm cartilage by contributing to the release of inflammatory cytokines and MMPs [44,45]. Sulfide is unequivocally accepted to ameliorate cartilage destruction. Such an effect was demonstrated in rat and human osteoarthritic samples [46,47,48,49,50]. Diallyl sulfide is a compound structurally similar to DMTS, albeit not a polysulfide. Diallyl sulfide reduced the release of MMP1, 3, and 13 from IL-1β-stimulated rabbit chondrocytes, and alleviated cartilage damage as well [51]. 

## 4. Materials and Methods

### 4.1. Patch Clamp

Whole-cell currents of voltage-clamped cells were recorded through manual patch-clamp electrophysiology according to standard protocols using Axopatch 200B amplifiers connected to a computer and digitized with Digidata 1550A (Molecular Devices, San Jose, CA, USA). GFP-positive *TRPA1* transfected CHO (Chinese hamster ovary) cells were identified using a Nikon Eclipse TE2000-U fluorescence microscope (Auro-science LLC, Budapest, Hungary). Pipettes were pulled from GC 150F-15 borosilicate glass capillaries (Harvard Apparatus, Holliston, MA, USA) in five stages with 4–10 MΩ resistance. Immediately before the measurement, the cells were maintained in the recording petri dish in a bath solution that consisted of 145 mM NaCl, 5 mM KCl, 1 mM MgCl_2_, 2.5 mM CaCl_2_, 5.5 mM glucose, and 10 mM HEPES (pH 7.35; 302–308 mOsmol/kg). For the recordings, the composition of the solution used in the patch pipette (internal solution) and in the perfusion system (external solution) was 150 mM NaCl, 10 mM HEPES, and 2 mM EDTA-Na (Ca^2+^-free solution; pH 7.35; ~300 mOsmol/kg). The solution exchange was achieved by using a gravity-flow system with continuous excess fluid removal. Peak currents were measured during 100 ms voltage ramps from 0 mV to +50 mV using a holding potential of 0 mV every 5 sec; data were acquired with pClamp10.7 (Molecular Devices, San Jose, CA, USA). In general, currents were low-pass-filtered using the built-in analog four-pole Bessel filters of the amplifiers and sampled at 5 kHz. Before analysis, whole-cell current traces were digitally filtered (five-point boxcar smoothing).

#### Data Analysis

Clampfit 10.7 (Molecular Devices, San Jose, CA, USA) and GraphPad Prism 5 (Graphpad, San Diego, CA, USA) were used for data display and analysis. For constructing the dose–response curve (DRC), the positive peak current (at +50 mV) was used. Current activation was quantified by the following equation:

$$\frac{I_{\mathrm{DMTS}\;\mathrm{peak}}-I_{\mathrm{base}}}{I_{\mathrm{AITC}\;\mathrm{peak}}-I_{\mathrm{base}}}=\frac{I_{\mathrm{DMTS}}}{I_{\mathrm{AITC}}}$$

where the baseline current (I_base_) is the peak current measured before the addition of the DMTS, I_DMTS_ is the peak current measured in the presence of DMTS at the indicated concentration, and I_AITC_ is the peak current measured in the presence of 100 µM AITC (allyl isothiocyanate), a known activator of TRPA1. For the fitting of the DRC, the Hill equation was used:

$$\frac{I_{\mathrm{DMTS}}}{I_{\mathrm{AITC}}}=\frac{X^{h}}{\mathrm{EC}50^{h}+X^{h}}\;$$

where X is the concentration of DMTS, h is the Hill coefficient, and EC50 is the median effective concentration.

### 4.2. Animals

*TRPA1* gene knockout (KO) and wild-type (WT) mice on a C57BL/6J background were used [52]. The mice were bred in the Laboratory Animal House of the Department of Pharmacology and Pharmacotherapy at the University of Pécs under standard pathogen-free conditions with food pellets and water available freely. The experiments ere harmonized with the 1998/XXVIII Act of the Hungarian Parliament on Animal Protection and Consideration Decree of Scientific Procedures of Animal Experiments (243/1998), the European Communities Council Directive of 2010/63/EU, and the requirements of the International Association for the Study of Pain (IASP). The experiments were approved by the Ethics Committee on Animal Research of the University of Pécs (license number BA02/2000-30/2016, granted 24 October 2016). The animals were sacrificed using cervical dislocation at the end of experiments. 

### 4.3. Induction of Serum-Transfer Arthritis and Treatment with DMTS

Arthritis was induced by a single injection of K/BxN serum (kindly donated by Attila Mócsai, Department of Physiology, Semmelweis University, Budapest; 300 μL i.p.). The control animals received nonarthritogenic BxN serum. DMTS (Sigma-Aldrich, Budapest, Hungary) solutions were prepared as described earlier [27]. The stock solution was prepared in dimethyl sulfoxide (Reanal, Budapest, Hungary; 1 mol/L), and was diluted 10× with saline containing polysorbate 80 (0.9% m/v NaCl and 2% v/v polysorbate 80; 100 mmol/L DMTS). The resulting solution was diluted with saline to 12.5 mmol/L. The vehicle contained dimethyl sulfoxide instead of DMTS. The animals were treated with DMTS (125 µmol/L, 10 mL/kg, i.p.) or the vehicle daily for 7 days. The solutions were prepared daily. Data on paw swelling, arthritis score, fluorescent imaging, and histology were obtained from the same 4 independent experiments. A further 4 independent experiments were performed for fluorescent imaging. These experiments did not involve the detection of other parameters. 

### 4.4. Measurement of Paw Smwelling

The volume of hind paws was measured using plethysmometry (Ugo Basile, Gemonio, Italy). The method required handling of the animals. The mice were habituated to the experimenter and instrument on 3 occasions before the administration of the K/BxN serum. Plethysmometry was performed 5 and 7 days after the serum injection. 

### 4.5. Arthritis Severity Score

Clinical appearance and function of the hind paws were scored by observers blinded to the treatments of the animals. Edema and redness of the hind legs, as well as passive mobility of the tibiotarsal joints, were considered. Scoring was performed on days 5 and 7 after serum injection. The score indicated the following conditions: 0–1.5, healthy hind leg; 1.5–2.5, minimal signs of inflammation; 2.5–4, mild arthritis; 4–7, moderate inflammation; and 7–10, severe arthritis [53,54]. 

### 4.6. Detection of the Rate of Plasma Extravasation by Fluorescent Imaging

The rate of plasma extravasation was detected before injection, as well as on days 2 and 6 after K/BxN serum injection, in order to avoid overlaps with testing of the mechanical pain threshold, paw, swelling, grip performance, and clinical scoring. The fluorescent dye IR676 was used. The solution contained Kolliphor HS 15 (5% *v*/*v*) to reach a particle size suitable for plasma-leakage detection. The animals were anesthetized with ketamine (120 mg/kg i.p.) and xylazine (12 mg/kg i.p.). The mice were then administered IR676 (0.5 mg/kg) into the retrobulbar venous plexus. Fluorescent imaging was performed 20 min after IR676 injection. An IVIS Lumina II (PerkinElmer, Waltham, MA, USA) was used with the following settings: auto acquisition time, F/stop = 1, binning = 2, excitation/emission filter 640/700 nm. The Living Image^®^ application (PerkinElmer, Waltham, MA, USA) was used for the evaluation of images. Fluorescence was expressed as total radiant efficiency = (photons/s)/(μW/cm^2^) [55]. 

### 4.7. Histological Analysis of Arthritic Tibiotarsal Joints

Tibiotarsal joints were harvested 7 days after the administration of the K/BxN serum. Joints were fixed in paraformaldehyde (4%, buffered). The samples were decalcified to enable the slicing of bones. Paraffin-embedded sections (3–5 μm thickness) were stained with hematoxylin and eosin, and the slides were scored by a blinded expert. The following parameters were scored on a scale of 0-3: cartilage destruction, mononuclear cell infiltration, synovial cell proliferation, and fibroblast number together with collagen deposition [55]. 

## 5. Conclusions

In conclusion, we demonstrated that DMTS allayed paw swelling and plasma extravasation in the K/BxN serum-transfer arthritis independently from the TRPA1 ion channel. Furthermore, DMTS exerted a chondroprotective effect and affected FLS cells. These actions require further investigation. Based on our data, DMTS and similar substances might become useful complementary therapeutics in RA.

## Data Availability

The data presented in this study are available upon request from the corresponding author.

## Supplementary Materials

The following supporting information can be downloaded at: https://www.mdpi.com/article/10.3390/ph15060671/s1: description of materials and methods regarding detection of mechanical pain threshold; measurement of grip performance; detection of myeloperoxidase enzyme activity by luminescent imaging. Figure S1: DMTS treatment had no effect on mechanical pain threshold of hind paws of *TRPA1* WT and KO mice, including groups treated with K/BxN serum, as well as ones receiving control BxN serum; Figure S2: DMTS treatment did not influence hanging performance of *TRPA1* WT and KO mice, including groups treated with K/BxN serum, as well as ones receiving control BxN serum; Figure S3: DMTS treatment did not affect myeloperoxidase activity in tibiotarsal joints of hind legs of arthritic *TRPA1* WT and KO mice detected 2 and 6 days after the inflammatory challenge; Figure S4: Number of infiltrating mononuclear cells in the tibiotarsal joints of hind legs of arthritic *TRPA1* WT and KO mice was unaffected by DMTS treatment; Figure S5: Synovial proliferation in the tibiotarsal joints of hind legs of *TRPA1* WT and KO animals injected with K/BxN serum was unaffected by DMTS treatment.
