# Supplementary material for: Investigation of the Role of the TRPA1 Ion Channel in Conveying the Effect of Dimethyl Trisulfide on Vascular and Histological Changes in Serum-Transfer Arthritis
Source: Pharmaceuticals (Basel). 2022 May 27;15(6):671. doi: 10.3390/ph15060671 (PMC9229242; doi:10.3390/ph15060671)
Supplement: Supplementary file 1 [file pharmaceuticals-15-00671-s001.zip › pharmaceuticals-1679000-supplementary.pdf]

Supplementary material

Materials and Methods

Detection of mechanical pain threshold

Dynamic plantar aesthesiometry (Ugo Basile, Gemonio, Italy) was utilized to assess mechanical pain threshold of hind paws. Mice were put into the compartments of the instrument to get used to the environment. Three baseline measurements were performed on before induction of serum-transfer arthritis. Measurements were conducted 5 and 7 days after serum administration. The stimulator went from 0 to 10 g in 4 seconds.

Measurement of grip performance

Mice were placed on a metal mesh platform and the platform was gently turned upside down and lifted 30 cm above the workbench. Animals use both front and hind paws to suspend themselves. Usually the hind paws loose grip first followed shortly by the front ones. The time until mice fell to the bench was noted. The cut-off limit was 60 seconds. Mice were trained to the exercise 3 times before serum challenge. Measurements were taken 5 and 7 days after the initiation of arthritis.

Detection of myeloperoxidase enzyme activity by luminescent imaging

Myeloperoxidase activity was measured before, as well as 2 and 6 days after arthritis induction. Animals received luminol sodium salt (5-amino-2,3-dihydro-1,4-phthalazine-dione; 30 mg/mL in phosphate-buffered saline, 150 mg/kg i.p.) Mice were anesthetized by ketamine (120 mg/kg i.p.) and xylazine (12 mg/kg i.p.). Bioluminescence was measured 10 min after luminol administration using an IVIS Lumina II (PerkinElmer, Waltham, USA) with the following settings: 120-s acquisition, F/stop = 1, binning = 8. Living Image® application (PerkinElmer, Waltham, USA) was used for data analysis. Luminescence was expressed as total radiance = total photon flux/s) [31].

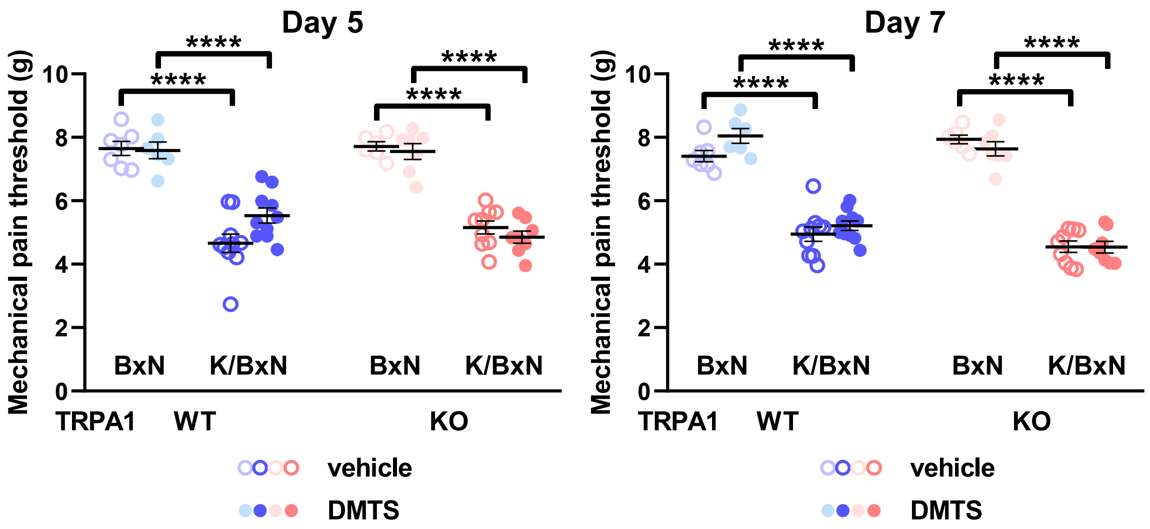

Supplementary Figure S1. DMTS treatment has no effect on mechanical pain threshold of hind paws of TRPA1 WT and KO mice including groups treated with K/BxN serum, as well as ones receiving control BxN serum. Mechanonociceptive tests werer performed 5 and 7 days after the initiation of arthritis. Mechanical pain threshold was measured by dynamic plantar aesthesiometry. Symbols indicate individual data points. Open symbols represent vehicle-treated animals, while solid symbols illustrate DMTS-treated ones. Horizontal lines indicate mean and whiskers show SEM. One-way ANOVA and Holm-Sidak's test, n = 6-10. **** p<0.0001 vs. indicated group.

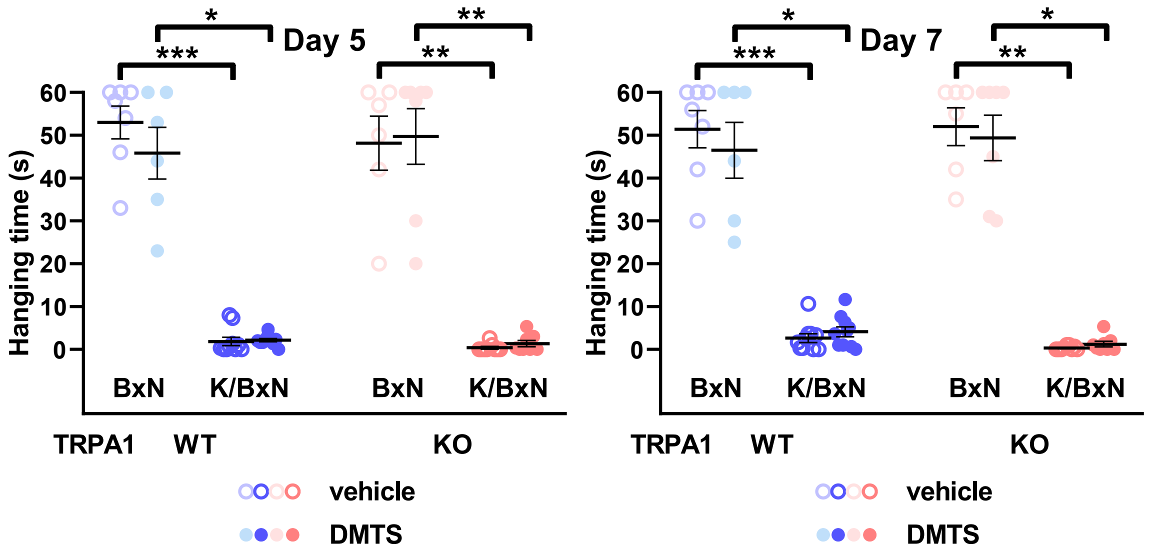

Supplementary Figure S2. DMTS treatment does not influence hanging performance of TRPA1 WT and KO mice including groups treated with K/BxN serum, as well as ones receiving control BxN serum. Mechanonociceptive tests werer performed 5 and 7 days after the initiation of arthritis. Mechanical pain threshold was measured by dynamic plantar aesthesiometry. Symbols indicate individual data points. Open symbols represent vehicle-treated animals, while solid symbols illustrate DMTS-treated ones. Horizontal lines indicate mean and whiskers show SEM. One-way ANOVA and Holm-Sidak's test, n = 6-10. **** p<0.0001 vs. indicated group.

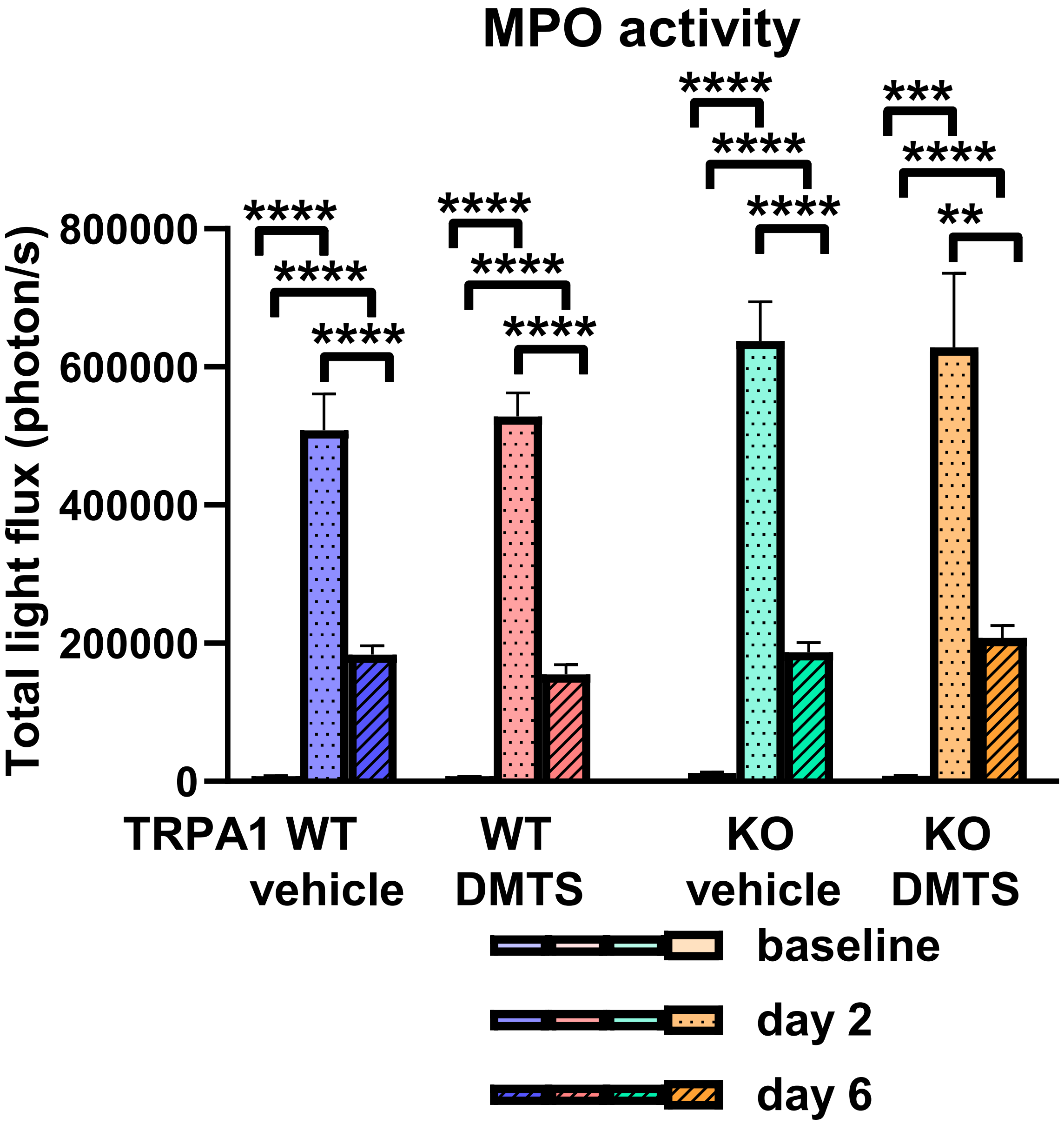

Supplementary Figure S3. DMTS treatment does not affect myeloperoxidase activity in tibiotarsal joints of hind legs of arthritic TRPA1 WT and KO mice detected 2 and 6 days after the inflammatory challenge. TRPA1 KO vehicle-treated mice exhibited larger myeloperoxidase activity on day 2 than their WT counterparts. Myeloperoxidase activity was detected by luminescent imaging using luminol. Solid bars represent baseline values, dotted bars show fluorescence at day 2 and striped bars indicate day 6. One-way ANOVA and Holm-Sidak's test, n = 14-20. * p<0.05, ** p<0.01, *** p<0.001, **** p<0.0001 vs. indicated group.

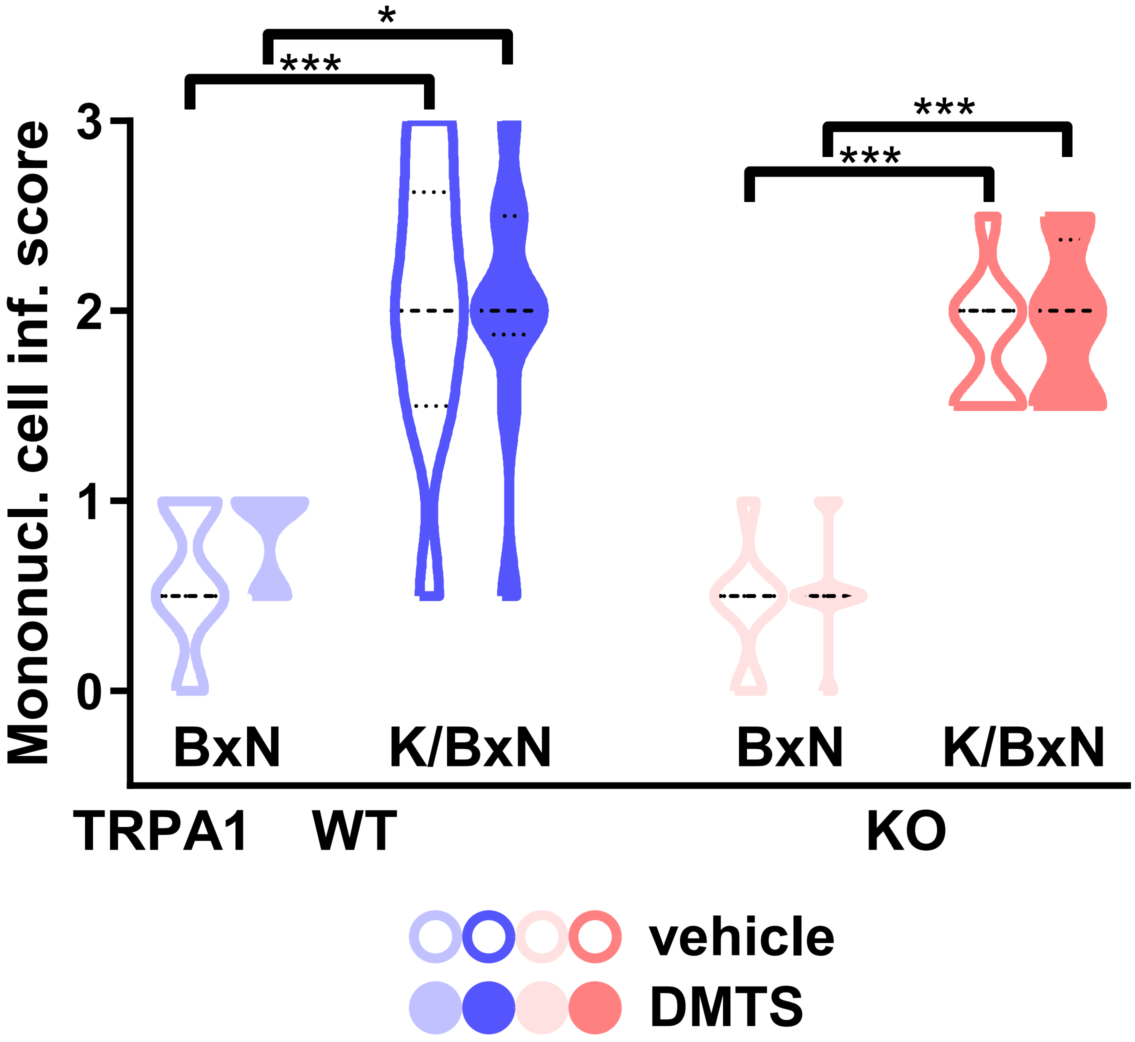

Supplementary Figure S4. Number of infiltrating mononuclear cells in the tibiotarsal joints of hind legs of arthritic TRPA1 WT and KO mice is unaffected by DMTS treatment. Number of mononuclear cells was scored on decalcified hematoxylin and eosin-stained slides. Width of violin plots hints frequency density of the data. Open violin plots represent vehicle-treated groups, solid ones indicate animals administered DMTS. Dashed lines show medians and dotted lines indicate quartiles. Kruskal-Wallis-test followed by Dunn's test, n = 8-10. * p<0.05, *** p<0.001 vs. indicated group.

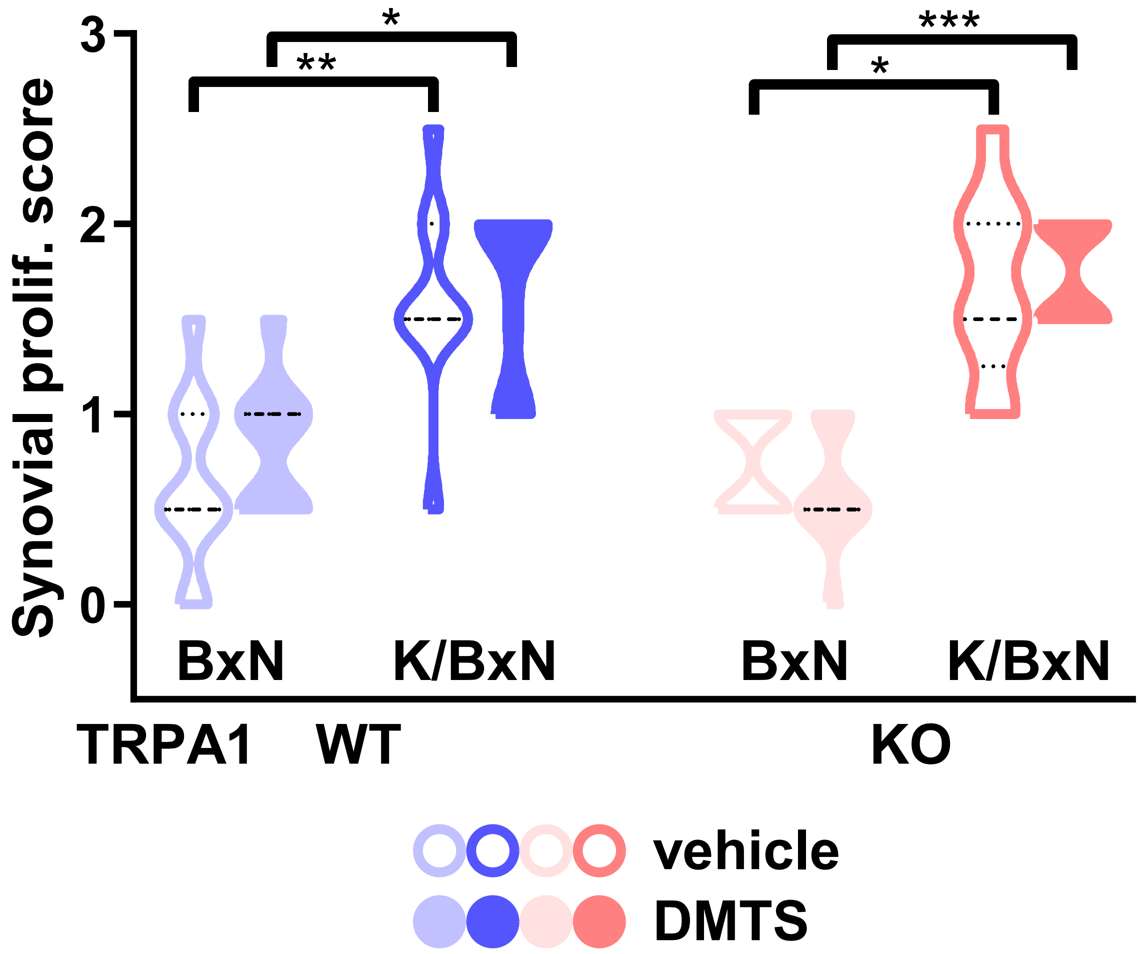

Supplementary Figure S5. Synovial proliferation in the tibiotarsal joints of hind legs of TRPA1 WT and KO animals injected with K/BxN serum is unaffected by DMTS treatment. Synovial proliferation was scored on decalcified hematoxylin and eosin-stained slides. Width of violin plots represents frequency density of the data. Open violin plots indicate vehicle-treated groups, solid ones show animals injected with DMTS. Dashed lines denote medians and dotted lines mark quartiles. Kruskal-Wallis-test followed by Dunn's test, n = 8-10. * $p<0.05$, ** $p<0.01$, *** $p<0.001$ vs. indicated group.
